# Dealing with heavy menstrual bleeding in very rare bleeding disorders: from a patient’s perspective

**DOI:** 10.1016/j.rpth.2026.103368

**Published:** 2026-01-23

**Authors:** Priyanka Raheja, Rezan Abdul-Kadir, Maja Johanne Søndergaard Knudsen, Christina Burgess, Saskia E.M. Schols, Very Rare Coagulation Factor Deficiency Working Group and Women and Girls with Bleeding Disorders Working Group of European Association for Haemophilia and Allied Disorders (EAHAD)

**Affiliations:** 1 Department of Haematology & Haemophilia Centre, The Royal London Hospital Haemophilia Centre, Barts Health NHS Trust, London, United Kingdom; 2 Department of Obstetrician and Gynaecology, The Royal Free NHS Foundation Hospital, Institute for Women's Health, University College London, London, United Kingdom; 3 Patient representative of the European Rare and Inhibitor Network, European Haemophilia Consortium, Brussels, Belgium; 4 Department of Clinical Microbiology, Copenhagen University Hospital – Amager and Hvidovre, Hvidovre, Denmark; 5 Haemophilia and Bleeding Disorders Counselling Association, Cambridge, United Kingdom; 6 Department of Haematology, Haemophilia Treatment Centre Nijmegen-Eindhoven-Maastricht, Radboud University Medical Centre, Nijmegen, The Netherlands

**Keywords:** hemostasis, heavy menstrual bleeding, patient perspectives, quality of life, rare bleeding disorder

## Abstract

Data on heavy menstrual bleeding in women with very rare bleeding disorders, such as very rare coagulation factor deficiencies or severe inherited platelet disorders, are scarce. Heavy menstrual bleeding is a common health problem in premenopausal women, causing both physical and psychosocial challenges. For women with very rare bleeding disorders, these challenges are unimaginably overwhelming, from hurdles during the diagnostic trajectory, facing regular monthly blood loss, to finding recognition of their unique position in life. Providing these women with a platform to share their life stories with their own true perspectives will increase awareness of this global health problem. In this opinion paper, we report the personal stories of 3 women living with very rare bleeding disorders and provide a future perspective on improving the quality of life for this population.

## 1 Introduction

Heavy menstrual bleeding (HMB) is a health problem with a high prevalence in the healthy population, up to nearly 50% [1]. This frequency leads to major challenges that women with a very rare bleeding disorder encounter: timely diagnosis of the congenital bleeding disorder and recognition of the impact of HMB on daily life. In this paper, we define women as individuals who menstruate. Studies and opinion papers have recently been published on the impact of HMB in women and girls with congenital bleeding disorders, emphasizing that early recognition and optimal management are crucial [[2], [3], [4], [5]]. As von Willebrand disease (VWD) is the most prevalent congenital bleeding disorder, most data on HMB have been collected from women with VWD. However, HMB is commonly observed in women with bleeding disorders of unknown cause and often leads to iron deficiency [6,7]. In this population, the important message is that management of HMB requires close collaboration among women, (pediatric) hematologists, gynecologists, and specialist nurses [8]. In a prospective clinical trial, almost all women with VWD and HMB perceived limitations in their overall life activities due to their menstrual period [9]. This negative impact on health-related quality of life was also shown in women with bleeding disorders of unknown cause [7].

In contrast to women with hemophilia and VWD, data on HMB in women with very rare bleeding disorders, such as very rare coagulation factor deficiencies and rare congenital platelet disorders, are scarce. In this paper, very rare coagulation factor deficiencies are defined as deficiencies of fibrinogen, factor (F)II, FV, combined FV and FVIII, FVII, FX, FXI, and FXIII. Rare congenital platelet disorders include M. Glanzmann and Bernard–Solier syndrome (harboring a biallelic genotype). In a Dutch nationwide cohort study of patients with a very rare coagulation factor deficiency, nearly 74% of included women experienced HMB since menarche, of which 82% did not ever receive any form of treatment [10]. The most striking outcome of this retrospective study was the enormous delay in diagnosis of the bleeding disorder, as the median age at diagnosis was 28 years, despite the presence of HMB since menarche. Another study, which combined data from 3 cohorts of congenital bleeding disorders (hemophilia, VWD, and very rare coagulation factor deficiencies), focused on gender differences with respect to the indication of diagnosis, bleeding phenotype, and management [11]. This study revealed that women with a congenital bleeding disorder were referred to a hematologist more often because of a bleeding diathesis (instead of a family history), had a longer diagnostic delay, and often required specific treatment for gender-specific bleeding (menstrual period, pregnancy, or childbirth) [11].

Considering the pronounced diagnostic delay, women with very rare coagulation factor deficiencies ultimately face an increased burden of disease and impaired quality of daily life [12]. This forum paper focuses on patients’ perspectives while dealing with HMB and its impact on quality of life in order to raise awareness of this health problem among women.

## 2 Patient Perspectives

**Figure 1:**
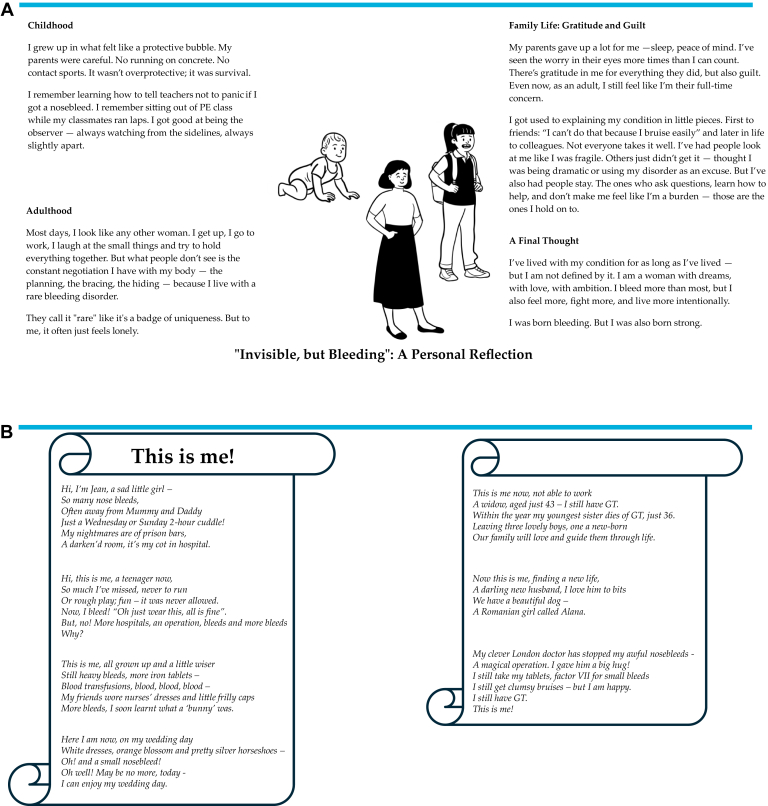
Patients’ personal stories of how to live with a very rare bleeding disorder from the early stages of life. (A) Perspective of a patient with Glanzmann thrombasthenia. (B) A personal poem on living with a very rare bleeding disorder.

To fully understand and recognize the impact of HMB on personal life, 3 women with a very rare bleeding disorder have shared their own stories (see also Figure 1A, B).

### 2.1 Patient story

I have a rare bleeding disorder called severe factor X deficiency, diagnosed when I was only one year old. Since then, my life has been shaped by the ups and downs of living with a chronic, often misunderstood condition, especially in a country like Lebanon where healthcare access is constantly disrupted by political and economic instability.

I had my first period at 15, and it was so heavy that I needed a blood transfusion. Since then, I’ve been on oral contraceptives (OCPs) to manage the heavy menstrual bleeding, but even with that, I still experienced episodes of very heavy periods over the years. It’s never been completely under control.

In terms of treatment, it’s been a long and difficult road. For years, the only thing available to treat bleeds was fresh frozen plasma, because prothrombin complex concentrates (PCCs) weren’t available in Lebanon. That changed briefly in 2017 when PCC was introduced — it was the first time I had access to a more effective, targeted therapy. With the collapse of the Lebanese economy, access to PCC disappeared, and I had no choice but to go back to fresh frozen plasma. It’s frustrating to know that the right medication exists but is simply out of reach because of where you live.

Over the 17 years since my first period, I’ve tried different OCPs, depending on what was available. I even tried to insert a hormonal intra uterine device, but it fell out just two weeks later. At one point, I had to undergo several surgical procedures to rule out endometriosis because my periods were so heavy and painful.

During school years, when the OCP worked, I could live more or less normally — but not always. The bleeding was often disabling. I missed a lot of school days, couldn’t participate in sports or any physical activity that involved risk of falling or injury. I remember constantly checking my pad for clots, worrying if I had leaked or not. Socially, it was limiting. I had to plan everything around the bleeding — or the fear of it.

Being anemic became routine. I’ve had to take iron supplements regularly, and when things got worse, I had intravenous iron to help manage the fatigue and consequences of low hemoglobin. I think there needs to be much more awareness — in schools, in the workplace — about rare bleeding disorders. I’ve spent most of my life finding excuses and covering up my symptoms, just to avoid being seen as weak or unreliable. What I need is not pity, but access to medication and a supportive environment so I can live, work, and hopefully become a mother like anyone else.

Looking ahead, my biggest concern is fertility. I want to get pregnant one day, but I worry about how my condition will affect it, and whether I’ll be able to carry a pregnancy safely. Another fear is transmitting this disorder to my children — it’s something I think about a lot. The uncertainty is hard, but I try to focus on what I can control: managing my health, staying informed, and hoping for better access to the care I need.

## 3 Future Perspective

Women with very rare bleeding disorders often face unique psychological challenges because their conditions are poorly understood, even within the medical community. When they feel unheard or dismissed, the impact can be significant. People with very rare bleeding disorders invariably experience great isolation, which can have a major impact on their mental well-being [12]. They are frequently the only person in their family with this very rare disorder, which can lead to a lack of understanding, even from family members. Added to this is the fact that they may never have met another person with the same disorder.

All the issues they may experience from establishing the right diagnosis to eventually receiving treatment create a difficult situation for this cohort across all genders. The issues women and girls have to face, such as HMB, fertility problems, childbirth, perimenopause, and menopause itself, have an almost incalculable impact on their quality of life.

However, the frustration and anger stemming from not being understood in the healthcare setting could lead to an erosion of trust in healthcare providers. Therefore, it is important to validate patients’ experiences and ensure that they feel heard in the healthcare setting. A knowledgeable, multidisciplinary team with experience in very rare bleeding disorders is more attuned to the unique issues faced by this population. The team should be encouraged to pay closer attention when a patient raises a psychological or psychosocial issue, and psychological care and peer support should be key parts of their care. Care providers should focus on addressing their worries, fears, and concerns about the future, and on sharing knowledge about potentially novel upcoming therapies. The impact on their physical and mental health, as well as their quality of life, should be addressed in the clinic. Recently, the retrospective experience of a Dutch multidisciplinary young women’s clinic for HMB was published [13]. The results revealed that 21% (32/153) of young women (aged ≤25 years) referred to this outpatient clinic suffered from a congenital bleeding disorder, with VWD and platelet function disorders being the most prevalent. First-line therapy to treat HMB was effective in alleviating blood loss in 30% of women, consisting of tranexamic acid with or without a combined OCP [13]. Iron deficiency was present in 52% (86/166) of women, leading to iron-deficient anemia in 28% (46/166) of those who needed iron supplementation. Unfortunately, no data on the quality of life of these young women were measured. In future studies, it would be beneficial to investigate how accurate diagnosis and access to treatment impact the quality of life in women with very rare bleeding disorders. With reference to the delay in diagnosis in patients with very rare coagulation factor deficiencies, we would expect differences in quality of life before and after diagnosis. While first-line therapy of HMB was effective in 30% of women in the aforementioned Dutch study [13], for some of the very rare bleeding disorders, no effective targeted treatment exists. For others, even when treatment exists, patients struggle to access it due to various factors, including economic barriers, as highlighted in the patient’s story.

In conclusion, the quality of life of women living with very rare bleeding disorders and suffering from HMB is impacted by both their physical and mental health. Physical health can be improved by eliminating delays in diagnosing very rare bleeding disorders, increasing awareness among frontline healthcare providers, and providing access to effective and available targeted treatment. For some of the very rare bleeding disorders, this requires research and development of affordable novel therapies. To break through the inevitable hurdle of isolation that ultimately comes from having a very rare disorder, patients should be provided with a forum at a national and international level to connect with other patients with a very rare bleeding disorder.
